# The adolescent transition under energetic stress: Body composition tradeoffs among adolescent women in The Gambia

**DOI:** 10.1093/emph/eot005

**Published:** 2013-04-09

**Authors:** Meredith W. Reiches, Sophie E. Moore, Andrew M. Prentice, Ann Prentice, Yankuba Sawo, Peter T. Ellison

**Affiliations:** ^1^Department of Human Evolutionary Biology, Harvard University, Cambridge, MA, USA; ^2^MRC Keneba, MRC Unit, The Gambia; ^3^MRC International Nutrition Group, London School of Hygiene and Tropical Medicine, London, WC1E 7HT, UK; and ^4^MRC Human Nutrition Research, Elsie Widdowson Laboratory, Cambridge, CB1 9NL, UK

**Keywords:** life history theory, puberty, body composition, reproductive ecology

## Abstract

**Background and objectives:** Life history theory predicts a shift in energy allocation from growth to reproductive function as a consequence of puberty. During adolescence, linear growth tapers off and, in females, ovarian steroid production increases. In this model, acquisition of lean mass is associated with growth while investment in adiposity is associated with reproduction. This study examines the chronological and developmental predictors of energy allocation patterns among adolescent women under conditions of energy constraint.

**Methodology:** Fifty post-menarcheal adolescent women between 14 and 20 years old were sampled for weight and body composition at the beginning and end of 1 month in an energy-adequate season and 1 month in the subsequent energy-constrained season in a rural province of The Gambia.

**Results:** Chronologically and developmentally younger adolescent girls gain weight in the form of lean mass in both energy-adequate and energy-constrained seasons, whereas older adolescents lose lean mass under conditions of energetic stress (generalized estimating equation (GEE) Wald chi-square comparing youngest tertile with older two tertiles 9.750, *P* = 0.002; GEE Wald chi-square comparing fast- with slow-growing individuals for growth rate 19.806, *P* < 0.001). When energy is limited, younger adolescents lose and older adolescents maintain fat (GEE Wald chi-square for interaction of age and season 6.568, *P* = 0.010; GEE Wald chi-square comparing fast- with slow-growing individuals for interaction of growth rate and season 7.807, *P* = 0.005).

**Conclusions and implications:** When energy is constrained, the physiology of younger adolescents invests in growth while that of older adolescent females privileges reproductively valuable adipose tissue.

## Abstract (precis)

Young female adolescents under energetic stress gain lean mass and lose fat mass while older adolescents lose lean mass and maintain fat. These findings support the prediction of life history theory that adolescence represents a period of transition from investment in growth to investment in reproduction.

## BACKGROUND AND OBJECTIVES

Puberty is the transition from non-reproductive juvenility to reproductively capable maturity. In the terms of life history theory [1], puberty represents a transition in energy allocation: during the juvenile period, energy available beyond the requirements of maintenance is used for growth, as demonstrated by accelerated growth rates in well-nourished populations relative to energy-constrained populations [2]. At puberty, this surplus energy begins to be invested in reproductive function [3]. For human females, reproductive function is reflected by ovarian steroid production. Ovarian estradiol promotes the conversion of energy into adipose tissue [4], which is mobilized during gestation and lactation [5]. In the adolescent female body, therefore, acquisition of lean mass, comprising bones, muscle, water and organs, equates, in life history terms, to investment in growth, while acquisition or preferential maintenance of adipose tissue can be understood as investment in reproduction.

Although puberty itself begins with a specific endocrine event, the initiation of pulsatile gonadotropin releasing hormone (GnRH) secretion from the arcuate nucleus of the hypothalamus [6, 7], the transition from juvenility to maturity occurs over the course of several years (Supplementary Fig. S1). Linear growth continues during this time, with height velocity in females generally peaking a year prior to menarche [8]. The overlap of these two phenomena, the adolescent linear height spurt and the externally visible sign of maturing gonadal function, indicates that adolescent physiology must allocate energy simultaneously to growth and reproductive function. This functional overlap is in keeping with the constrained fecundity seen in the years immediately after menarche, a phenomenon often referred to as ‘adolescent sterility’ but more accurately termed ‘adolescent subfecundity’ [9, 10].

The role of energy availability in mediating the timing of pubertal maturation in traditional and industrialized populations has been documented: while a high ratio of adult to juvenile extrinsic mortality risk promotes early age at maturity even when energy is limited [11, 12], there is generally a negative relationship between energy availability and juvenile growth rates on the one hand and age at puberty on the other hand [2]. Less is known, however, about the determinants of somatic energy allocation during puberty. This is a particularly relevant question for females, for whom a single reproductive event equates to 300 average kilocalories per day for the 9 months of gestation (calculated with the equation from Aiello and Key [13] for a 42.2 kg !Kung woman) and 640 kcal per day for 6 months of lactation [14].

At the same time, adiposity is not the only somatic reproductive asset in women: in some developing world populations, female height correlates positively with marriageability and with reproductive success [15–17], suggesting that somatic investments in linear growth—or in one of its correlates, such as pelvic growth—may yield reproductive dividends. It is important to keep in mind, however, that greater height may indicate that growth has already ceased and the individual is prepared to invest in reproduction.

This study investigated the determinants of somatic allocation strategy in energetically constrained adolescent women, many of whom have not completed linear growth. We asked the question: What developmental and chronological markers predict the transition from preferential investment in growth in the form of lean mass to investment in reproduction in the form of fat mass? We predicted that, in an energetically constrained population of adolescent women in The Gambia, developmental age would predict somatic energy allocation strategy. The answer to this question will contribute to our understanding of what constitutes evolutionarily relevant cues to modulating the tempo of reproductive maturation in females.

## METHODOLOGY

### Study subjects and field site

Participants were 67 adolescent females between 14 and 19 years at enrolment, born to mothers enrolled in a 1989–94 protein, energy and micronutrient supplementation trial conducted across the rural West Kiang Region of The Gambia and co-ordinated by the Medical Research Council (MRC) field station in Keneba, The Gambia. Half the mothers received pregnancy supplements from 20 weeks gestation until delivery whereas the other half received supplements from delivery for 20 weeks. Daily supplements were 4250 kJ and 22 g protein [18]. Subjects enrolled in this study were resident in West Kiang, a rural province of The Gambia where the highly seasonal environment consists of a hungry season (June to October) characterized by population weight loss and a harvest season in which weight is gained [19]. All daughters enrolled in this study were born in the hungry season, June through October inclusive, the time of year when the pregnancy supplement had the greatest impact on birth weight [18]. Participants were post-menarcheal, not pregnant, had reported at least one period since parturition if lactating and were not using hormonal contraception.

### Study design

Data reported here were collected in March during the 2010 harvest season and during a 30-day period spanning July and August in the 2010 hungry season. Anthropometric measurements, weight and body composition were measured at the beginning of each data collection period at MRC Keneba, using standard procedures with regularly validated equipment (see below). Weight and body composition were measured again at the end of the data collection period in participants’ villages. First morning fasting urine samples and non-fasted morning blood spots were collected approximately weekly at participants’ homes and transported to MRC Keneba laboratory for processing.

### Anthropometry

Height, weight and triceps skinfold thickness were measured in triplicate by the same trained observers. Height was measured in barefoot participants to the nearest millimeter with a stadiometer (Leicester height measure, Seca 214), calibrated daily with a wooden rod of known length. Weight was measured in light clothing in triplicate to the nearest 0.1 kg on a battery-operated scale (Tanita Corporation, Japan), placed on a level surface and calibrated daily with a 10-kg weight. Skinfold measures were taken to the nearest 2 mm with Holtain calipers (Holtain).

### Body composition

Body composition was measured with the Tanita BC-418MA segmental body composition analyzer at the beginning and end of each sampling season. Prins *et al.* [20] validated the Tanita inbuilt prediction equation estimates against total body water estimates of body composition in Gambian children and developed a population-specific equation for the estimation of percent fat-free mass. The correlation of this estimation equation with estimates from deuterium was *R* = 0.84 (95% CI 0.79–0.89) [20]. A modified version of this equation, which includes using triceps skinfold measures, was used to convert Tanita impedance readouts in Ohms into estimates of fat and lean mass [21].

### Biological samples

Please see Supplementary Materials for detailed collection and analysis methods for C-peptide of insulin and leptin.

### Statistical analysis

Statistical analysis was performed in IBM SPSS Statistics Version 19.0. GEEs were used to assess the effect of chronological age, gynecological age and height velocity on within-season changes in weight and body composition (fat mass and lean mass). Marginal means reflect uneven sample sizes in groups. Potential covariates included in the models were age, height and weight (for analyses of fat and lean mass). Results were considered significant at *P* < 0.05.

### Ethics

Ethics approval for the study in The Gambia was granted by the joint Gambia Government/MRC The Gambia Ethics Committee (proposal SCC 1169). Permission for Harvard personnel to conduct the study was granted at Harvard by the Committee for the Use of Human Subjects (application #F-17744-102).

## RESULTS

### Study subject characteristics

Chronological, developmental and anthropometric characteristics of study participants are detailed in Table 1. Because no effect of maternal treatment group was found in analysis, treatment group was not included as a term (data not presented). Of the 67 women enrolled, data for this analysis were available for 50. The other 17 women could not be located for end-of-month data collection. Differences in sample size between the harvest and hungry season are due to participants becoming pregnant, transferring out of the study area or withdrawing from the study.

**Table 1.** Participant characteristics by season

	Harvest season (mean ± SE), *n* = 47	Hungry season (mean ± SE), *n* = 29	Season Wald-chi square and *P-*value
Age (years)	17.30 (0.21)	17.98 (0.28)	5700, *P* < 0.001***
Gynecological age (years)	2.2 (0.2)	2.9 (0.3)	5710, *P* < 0.001***
Height (cm)	161.0 (0.8)	161.4 (1.2)	10.7, *P* = 0.001**
Start of season weight (kg)	52.7 (1.1)	55.8 (1.6)	40.2, *P* < 0.001***
End of season weight (kg)	52.6 (1.1)	54.9 (1.5)	6.29, *P* = 0.012*
Start of season % fat (Tanita derived)	21.8% (0.6)	24.0% (0.7)	67.9, *P* < 0.001***
End of season % fat (Tanita derived)	21.8% (0.6)	23.3% (0.8)	10.2, *P* = 0.001**
Log leptin (ng/ml)	1.1 (0.0)	1.0 (0.0)	4.32, *P* = 0.038*
Log C-peptide of insulin ng/creatinine (mg)	1.2 (0.0)	0.9 (0.1)	12.8, *P* < 0.001***

This table represents the subset of 50 individuals for whom beginning and end of season weight and body composition data are available. *P < 0.05. **P < 0.01. ***P < 0.001.

### Predictor variables

#### Age

Age refers to chronological age at the beginning of the study season. In the present analysis, the youngest tertile was compared with the group comprising the older two tertiles. This is because analysis revealed clear biological differences between those closer to the beginning of puberty and thus in the midst of pubertal growth relative to those who were nearer completion of growth and maturation processes.

#### Gynecological age

Gynecological age is years since menarche. Menarcheal age, as self-reported recall data, is prone to errors of memory [22], which are compounded, in this case, by differences between researchers’ and participants’ concepts of time. Nonetheless, three lines of evidence indicate that menarcheal ages reported here are biologically relevant. First, median age at menarche in the study population was 15.00 years (95% confidence interval 14.92–15.42), while a recent probit analysis of age at menarche in the same population found a similar median menarcheal age of 14.90 (95% confidence interval 14.52–15.28) [23]. Second, there was no significant variation in reported menarcheal age relative to date of birth: an ANOVA assessing age at menarche by year of birth was non-significant (*F* = 1.64, *P* = 0.16). Third, we would predict that developmentally younger individuals have greater height velocity. As expected, average height velocity across the study period was associated negatively with gynecological age (GEE estimated marginal means of height velocity for gynecological age tertiles 1.0 cm/year in the youngest group, 0.9 cm/year in the middle group and 0.7 cm/year in the oldest group; Wald chi-square for gynecological age by tertile 14.6, *P* = 0.001, *n* = 52). As with age, the youngest tertile was compared with the older two tertiles in GEE analyses.

#### Height velocity

Height velocity in cm/year was estimated in all individuals who were present for anthropometric measurement in at least two sampling seasons. Analysis compared individuals whose growth rate met or exceeded 1.0 cm/year, ‘fast growers’, with those whose growth rates were <1.0 cm/year, ‘slow growers’. Height velocity is a better proxy of maturity than height in the post-menarcheal period when age-related height differences are less important than final height differences.

#### Relationship among predictor variables

Chronological and developmental variables are correlated both biologically and statistically. Each is presented separately here because each tracks a slightly different biological process: while age marks the passage of time, with which the probability of maturational events increases, gynecological age signifies distance from a threshold reproductive event in an individual’s unique maturational history. Height velocity, meanwhile, eventually reaches zero in all individuals; a snapshot of height velocity therefore allows an estimate of how close to this predetermined endpoint a given adolescent may be.

Age and gynecological age tertiles were assigned across the full sample, of which a subset is represented in within-season weight and body composition measurements. Therefore, there were different numbers of individuals in the age and gynecological age tertiles. Overlap among age and gynecological age tertiles and height velocity categories in the harvest and hungry seasons is illustrated in Supplementary Fig. S2.

#### Differences in energy availability between harvest and hungry seasons

The current data indicate that harvest season was characterized by greater energy availability than the hungry season. Three lines of evidence demonstrate this difference. First, within season weight change in the study population was positive in the harvest season and negative in the hungry season (weight change in kg estimated marginal mean in the harvest season 0.2 (SE 0.2), hungry season −0.3 (SE 0.1), Wald chi-square of season 7.84, *P* = 0.005, age covariate Wald chi-square 7.47, *P* = 0.006, *β* = −0.210, *n* = 49).

Second, the population as a whole maintained fat mass in the harvest season and lost fat mass in the hungry season (fat mass change in kg estimated marginal mean in the harvest season 0.0 (SE 0.1), hungry season −0.4 (SE 0.1), Wald chi-square of season 12.0, *P* = 0.001).

Finally, leptin and C-peptide of insulin, endocrine markers of long- and short-term energy status, respectively, were significantly higher in the harvest season than in the hungry season (log leptin in ng/ml estimated marginal mean in the harvest season 1.1 (SE 0.0), hungry season 0.95 (SE 0.0), GEE Wald chi-square for season 39.6 *P* < 0.001, fat mass covariate Wald chi-square 104.5, *P* < 0.001, *β* = −7.107 × E^−^^6^, *n* = 53; log C-peptide ng/Cr mg estimated marginal mean in the harvest season 1.1 (SE 0.0), hungry season 0.93 (SE 0.0), Wald chi-square of season 11.9, *P* = 0.001, age covariate Wald chi-square 6.58, *P* = 0.01, *β* = −0.072, *n* = 52). Taken together, these results indicate that energy was limited in the hungry season relative to the harvest season, and the impact of energy constraint on weight and on C-peptide of insulin was greater in older individuals. Height and weight were not significant in any of the models and thus were not included as covariates.

#### Predictors of within-season weight change

**Figure 1.**
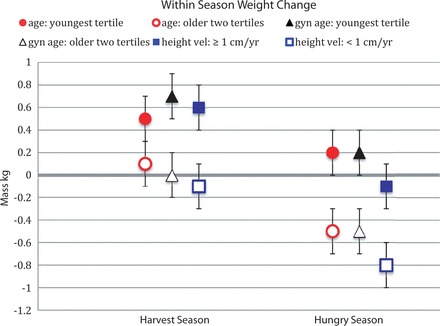
Within season weight change in the harvest and hungry seasons as predicted by age (*P* = 0.005), gynecological age (*P* = 0.002) and height velocity (*P* = 0.005). Wald chi-square statistics and additional *P*-values are in Table 2. Estimated marginal means and standard errors are in Table 3

On all measures of chronological and developmental age, younger and faster growing individuals gained more weight than older, slower growing individuals in both the harvest season and the hungry season (Fig. 1). (For this analysis and for those below, Wald chi-square statistics for factors and covariates are available in Table 2 and estimated marginal means of group differences are in Table 3.) Both the gynecological age and height velocity models confirmed the finding that weight gain was more positive in the harvest season (Table 2). Height at entry into the study was positively associated with weight gain when age was a predictor (Table 2). This finding does not necessarily contradict the association between youth and weight gain, because height and age were not correlated in the sample population (GEE ns).

**Table 2.** GEE Wald chi-square and *P-*values for analyses of age, gynecological age and height velocity as predictors of within-season change in weight and body composition

	Wald chi-square and *P-*values		
	Weight	Lean	Fat
Age			
Age	7.89, *P* = 0.005**	9.75, *P* = 0.002**	0.56, *P* = 0.453
Season	3.53, *P* = 0.060	0.18, *P* = 0.672	19, *P* < 0.001***
Interaction	0.150, *P* = 0.699	4.255, *P* = 0.039*	6.568, *P* = 0.010*
Covariate and β	Height 3.95, *P* = 0.047*, *β* = 0.032	Height 4.71, *P* = 0.030*, *β* = 0.036	NS
Gynecological age			
Gynecological age	9.45, *P* = 0.002**	4.53, *P* = 0.033*	NS
Season	7.11, *P* = 0.008**	0.10, *P* = 0.750	NS
Interaction	0.01, *P* = 0.905	0.28, *P* = 0.599	NS
Covariate and β	NS	Weight 4.36, *P* = 0.037*, *β* = −0.025	NS
Height velocity			
Height velocity	7.79, *P* = 0.005**	19.8, *P* < 0.001***	0.67, *P* = 0.412
Season	8.68, *P* = 0.003**	3.16, *P* = 0.076	5.76, *P* = 0.016*
Interaction	0.03, *P* = 0.854	3.63, *P* = 0.057	7.81, *P* = 0.005**
Covariate and β	NS	NS	Age 4.26, *P* = 0.039*, *β* = −0.089

Age and gynecological age comparisons are between the youngest tertile and older two tertiles, while height velocity comparisons are between individuals growing ≥1 cm/year and those growing <1 cm/year. *P < 0.05. **P < 0.01. ***P < 0.001.

**Table 3.** Estimated marginal means and standard error for within-season weight change

	Weight, kg (SE)		Lean mass, kg (SE)		Fat mass, kg (SE)	
	Harvest	Hungry	Harvest	Hungry	Harvest	Hungry
Age						
Youngest tertile	0.5 (0.2)	0.2 (0.2)	0.4 (0.2)	0.9 (0.2)	0.1 (0.1)	−0.8 (0.2)
Older two tertiles	01 (0.2)	−0.5 (0.2)	0.2 (0.2)	−0.2 (0.2)	−0.1 (0.1)	−0.4 (0.1)
Gynecological age						
Youngest tertile	0.7 (0.2)	0.2 (0.2)	0.5 (0.2)	0.5 (0.2)	NS	NS
Older two tertiles	0.0 (0.2)	−0.5 (0.2)	0.1 (0.2)	0.0 (0.2)	NS	NS
Height velocity						
≥1 cm/year	0.6 (0.2)	−0.1 (0.1)	0.5 (0.2)	0.6 (0.2)	0.0 (0.1)	−0.6 (0.1)
<1 cm/year	−0.1 (0.3)	−0.8 (0.3)	0.0 (0.2)	−0.7 (0.2)	−0.1 (0.1)	0.0 (0.1)

Weight changes occur over a month. Harvest season *n* = 47. Hungry season *n* = 29.

#### Predictors of within-season lean mass change

**Figure 2.**
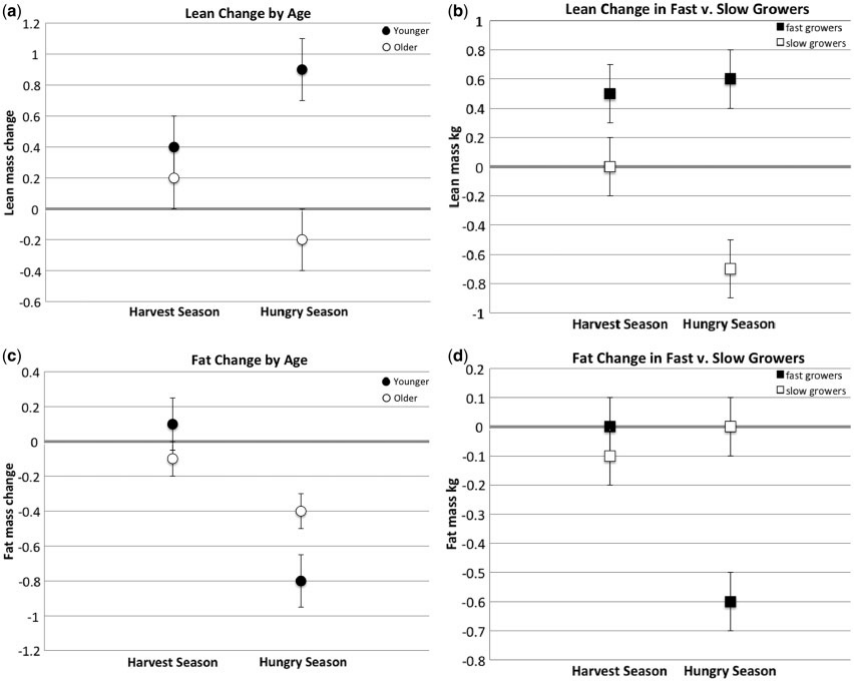
(**a**) Comparison of lean mass change in the harvest season and the hungry season in the youngest and older two tertiles (age *P* = 0.002, season NS, interaction *P* = 0.039). Wald chi-square statistics in Table 2; estimated marginal means in Table 3. (**b**) Comparison of lean mass change in the harvest season and the hungry season in individuals growing ≥1 cm/year relative to those growing <1 cm/year (height velocity *P* < 0.001, season NS, interaction NS). Wald chi-square statistics in Table 2; estimated marginal means in Table 3. (**c**) Comparison of fat mass change in the harvest season and the hungry season in the youngest and older two tertiles (age NS, season *P* < 0.001, interaction *P* = 0.010). Wald chi-square statistics in Table 2; estimated marginal means in Table 3. (**d**) Comparison of fat mass change in the harvest season and the hungry season in individuals growing ≥1 cm/year relative to those growing <1 cm/year (height velocity NS, season *P* = 0.016, interaction *P* = 0.005). Wald chi-square statistics and additional *P*-values in Table 2; estimated marginal means in Table 3

In both hungry and harvest seasons, younger and more rapidly growing individuals gained more lean mass than older and less rapidly growing individuals (Fig. 2 and Tables 2 and 3). The youngest tertile in gynecological age gained more lean mass than older tertiles in both seasons (Table 2). When participants were divided by age, older individuals lost lean mass in the hungry season whereas the youngest tertile did not (Tables 2 and 3). Similarly, slower but not faster growing individuals lost lean mass in the hungry season (Tables 2 and 3).

#### Predictors of within-season fat mass change

Chronologically younger individuals lost significantly more fat in the hungry season than in the harvest season (Fig. 2 and Tables 2 and 3). Although the mean fat change among older individuals in the hungry season was likewise negative, it was not significantly different from this group’s within-season fat change in the harvest season (Table 2). This pattern was echoed in height velocity groups: while fat mass remained constant for both fast and slow growers in the harvest season, the slow growers maintained fat mass in the hungry season while fast growers lost it (Fig. 2 and Tables 2 and 3). Gynecological age alone did not predict within-season fat mass change.

## CONCLUSIONS AND IMPLICATIONS

Data from the study sample indicate that, during the adolescent life history transition, the bodies of post-menarcheal adolescent women in The Gambia responded to energetic stress with somatic energy allocation strategies that appeared to differ by age and developmental stage. Those who were chronologically younger and gaining significant height preferentially acquired lean mass, both in a season of relative energy abundance and in a season of relative energy constraint, mobilizing adipose tissue during the hungry season. In contrast, adolescent women who had neared or reached the end of linear growth and those who were chronologically older lost lean mass during the hungry season. These results are consistent with earlier findings that pregnant women who are still growing allocate a higher proportion of energy to maternal relative to fetal tissue than do comparably aged non-growing pregnant women [24] (though see also [25]). The current findings are also consistent with life history theory, suggesting that shifts in somatic priorities of energy allocation occur progressively during puberty. Given that all participants were post-menarcheal and height velocities were low across the sample, it is possible that differences in intra-somatic allocation strategy would be even more apparent in a sample including younger adolescents.

When height velocity alone was considered, it appeared that slower growing adolescent women mobilized lean mass and preserved fat mass, suggesting that ovarian function in this subset of individuals may have been more robust than in faster growing women, with higher estradiol levels promoting maintenance of adipose depots important to gestation and lactation. Additional research will be needed to establish where on the body adipose tissue is preferentially maintained in developmentally and chronologically older adolescents under conditions of energy constraint. The authors hypothesize that gluteofemoral adipose depots will be favored, as these reserves have been shown to support gestation and lactation [26].

The findings reported here differ from and contribute to previous research in significant ways. Although cross-sectional patterns in female body composition with age and parity have been reported [27], short-term longitudinal shifts in intra-somatic allocation among fat and lean mass during puberty have not. Prior analyses of body composition in non-pregnant, non-lactating Gambian women found preferential mobilization of fat tissue during the hungry season in study participants 20–35 years of age [28], indicating that the result reported here may be specific to the pubertal period. Second, we did not detect energy sparing in activity during the hungry season relative to the harvest season, in contrast to data from a similar population in Senegal [29].

One finding that requires further exploration is the relative importance of height velocity compared with gynecological age in predicting within-season somatic energy allocation strategies in adolescent women. Specifically, why was height velocity important while distance from menarche appeared to be less salient as a determinant of fat mass change? We will consider two complementary explanations for the importance of height in this study, since prioritizing investment in linear growth suggests that height itself is biologically significant. (Recall that height and age were not correlated in the sample population, suggesting that younger, faster growing individuals were not merely approaching a height threshold but rather were investing in height on its own behalf, or on behalf of a physiologically relevant trait that correlates with height.)

The lesser explanatory power of gynecological age may be understood by keeping in mind two characteristics of pubertal maturation: first, menarche represents not a point of biological transition, as from sterility to fecundability, but rather a threshold at which the functioning of the hypothalamic–pituitary–ovarian axis becomes visible. Follicular estradiol production has reached a level at which the products of endometrial lining proliferation can no longer be resorbed and must be shed [30]. This proliferation, however, is no guarantee that ovulation has occurred [9]. The relationship of menarche to somatic energy allocation, therefore, is not completely clear: while there must be pubertal levels of circulating estradiol for menarche to occur, estradiol contributes both to reproductive function and to linear growth [31], making its relative importance in these two processes at the adolescent threshold point indeterminate. Differences in the relationship of height velocity, final height and menarche in resource-constrained and resource-abundant populations affirm the variability of the menarche-growth relationship [11]. Second, menarche typically occurs about a year following peak height velocity [8]. Given that there is inter-individual variation in the magnitude and duration of the pubertal spurt in linear growth (Supplementary Fig. S3), gynecological age or distance from menarche may correspond to different points in the individual’s linear growth trajectory [32]. That growth trajectory itself may be more theoretically and practically robust as a predictor of somatic energy allocation.

Second, height velocity may be a signal of structural maturation that is easy to measure outwardly and that serves as a visible indicator of other dimensions of skeletal maturation, such as remodeling of the interior dimensions of the bony pelvis in females, which, even more than increases in bi-iliac breadth or hip circumference, is the axis of anatomical change most important to successful parturition in humans [33]. A related and not mutually exclusive possibility is that height is one marker of reproductive value in resource-scarce ecologies, perhaps constituting a metric of the quality of the individual’s developmental environment and thus the somatic resources that she will be able to invest in reproduction [3]. Height associates positively with marriageability [15] and reproductive success [16] in many non-Western populations, including The Gambia [17], though the significant outcome measure in the Gambian population is not number of births but survival of offspring, indicating that women of different heights may be able to allocate different amounts of energy to fetal or infant growth or immune function.

It is worthwhile to note that there was strong evidence of growth and maturation in height, weight and adiposity in the study population across seasons (Table 1), even as within-season changes in weight and fat mass tended to be negative in the hungry season. The shorter term patterns detected through within-season analysis revealed responsiveness to energetic stress that was not discernible from data collected at less frequent intervals. Subtle seasonal changes in weight and body composition of the kind documented here likely reflect the type of facultative shifts in somatic energy allocation that conferred a selective advantage over the course of human evolution: the body must not only invest available resources in the most beneficial life history category, growth or reproduction, but it must prioritize which type of somatic store to preserve and which to mobilize when energy is limiting. The ability to negotiate these tradeoffs adaptively during the pubertal transition is necessary to acquiring the somatic capital that underwrites reproduction while taking advantage of reproductive opportunities at energetically favorable moments.

In considering these results it is important to note that they focus on individuals in the 15–20-year age range who are not pregnant or in lactational amenorrhea. It is possible that there were physiological differences in somatic energy allocation strategy between this group and their age- and size-matched peers who were pregnant or in lactational amenorrhea and thus were not eligible for inclusion in this study. Additional limitations include unequal sample sizes in hungry and harvest seasons and a relatively small overall sample size.

In summary, changes in weight and body composition over the course of an energetically constrained hungry season and a less energetically constrained harvest season indicated that chronologically and developmentally younger adolescent women preferentially allocate somatic resources to growth, while their older and more developed peers preserve reproductively valuable adipose tissue when energy is limiting. These results support an understanding of adolescence as a period of life history transition from juvenile growth to mature reproductive investment. The importance of height velocity as a predictor of somatic allocation strategy underscores its status as proxy for the degree to which the growth period is complete and the reproductive period begun.

## SUPPLEMENTARY DATA

Supplementary data is available at *EMPH* online.

## FUNDING

The research was supported by a Doctoral Dissertation Improvement Grant (BCS-0925768) and by a Senior Research Grant (BCS-0921237) from the National Science Foundation of the USA. This work was supported by the UK Medical Research Council under program numbers MC-A760-5QX00, U105960371 and U123261351.

**Conflict of interest**: None declared.

## Supplementary Material

Supplementary Data
