# Supplementary material for: The adolescent transition under energetic stress: Body composition tradeoffs among adolescent women in The Gambia
Source: Evol Med Public Health. 2013 Apr 9;2013(1):75–85. doi: 10.1093/emph/eot005 (PMC3868354; doi:10.1093/emph/eot005)
Supplement: Supplementary Data [file supp_2013_1_75__index.html]

The adolescent transition under energetic stress — Supplementary Data 

# The adolescent transition under energetic stress

## Supplementary Data

files

**Files in this Data Supplement:**

- Supplementary Data - docx file
- Supplementary Data - docx file
- Supplementary Data - docx file
- Supplementary Data - docx file
