# Supplementary material for: The adolescent transition under energetic stress: Body composition tradeoffs among adolescent women in The Gambia
Source: Evol Med Public Health. 2013 Apr 9;2013(1):75–85. doi: 10.1093/emph/eot005 (PMC3868354; doi:10.1093/emph/eot005)
Supplement: Supplementary Data [file supp_eot005_Reiches_et_al_EMPH_Supplemental_Methods.docx]

*Biological samples*

C-peptide of insulin

C-peptide of insulin was measured in first morning void urine samples dried on saturated filter paper (Whatman 903 Biological and ID-Biological Perkin Elmer 226, which have comparable recovery rates). C-peptide of insulin was measured by competitive I125 radioimmunoassay (Human C-peptide RIA kit manufactured by Millipore, catalogue number HCP-20K). Additional assay buffer (catalogue number AB-P) was used to elute samples. Standard curve standards were 0.1, 0.2, 0.5, 1, 2, and 5 ng/ml. Five paper punches of each sample were incubated overnight at 4^o^C with 500 μl assay buffer, creating a 1:5 dilution. The following day, 50 μl sample aliquots were assayed according to kit instructions via the overnight protocol. Samples with C-peptide concentrationss lower than detectable limits were assayed again at double concentration. Samples with C-peptide concentrations higher than detectable limits were assayed again at half concentration. Creatinine corrections were applied to adjust for urinary concentration (Taussky 1954). The intra-assay coefficient of variation for the high control was 13.4% and for the low control was 13.8%. Inter-assay coefficients of variation were 10.1% for high and 15.7% for low controls (N=13 assays). The sensitivity of the assay was 0.01 ng/ml.

Leptin

Approximately 100 μl of peripheral whole blood was collected by finger prick into Microtainer capillary tubes. Samples were collected between 9h00 and 11h00 and were not fasted. On arrival at MRC Keneba, samples were allowed to separate before centrifugation for 20 minutes at 1000 rpm. Serum was pipetted into Starstedt microtubes. Samples were stored at -80^o^C until assay. Analysis of leptin stability in serum indicates that sample concentrations remain stable under this treatment regimen ([23](#_ENREF_23)). Serum leptin was analyzed in the laboratory at MRC Keneba in May 2010 (harvest season) and August 2010 (hungry season). A commercially available 96-well plate leptin sandwich ELISA kit with horseradish peroxidase-labeled antibody (BioVendor, Candler, NC) was used according to kit protocol. Calibration range was 1-50 ng/ml and the limit of detection was 0.17 ng/ml. The intra-assay coefficient of variation was 2.8% for the high control and 7.8% for the low control, and the inter-assay coefficient of variation was 16%for the high control and 50% for the low control (N=10). Output from the plate reader was analyzed by Ascent software.
