# Supplementary material for: The adolescent transition under energetic stress: Body composition tradeoffs among adolescent women in The Gambia
Source: Evol Med Public Health. 2013 Apr 9;2013(1):75–85. doi: 10.1093/emph/eot005 (PMC3868354; doi:10.1093/emph/eot005)
Supplement: Supplementary Data [file supp_eot005_Reiches_et_al_EMPH_Suppl_Fig_1.docx]

Supplementary Figure 1. Energy allocation to linear growth in the form of height velocity (dashed line) decreases as gonadal steroid production (solid line) increases. The two life history functions overlap during adolescence.

time

gonadal steroid production

height velocity
