# Supplementary material for: The adolescent transition under energetic stress: Body composition tradeoffs among adolescent women in The Gambia
Source: Evol Med Public Health. 2013 Apr 9;2013(1):75–85. doi: 10.1093/emph/eot005 (PMC3868354; doi:10.1093/emph/eot005)
Supplement: Supplementary Data [file supp_eot005_Reiches_et_al_EMPH_Suppl_Fig_2.docx]

Supplementary Figure 2. (a) Dry harvest season: number of individuals in each age and developmental group and overlap among groups.

Fast growers

n = 25

Gyn age youngest tertile

n = 18

Age

youngest tertile

n = 21

**2**

**5**

**2**

4

8

**10**

4

Supplementary Figure 2. (b) Rainy agricultural season: number of individuals in each age and developmental group and overlap among groups.

Fast growers

n = 18

**4**

9

8

7

**3**

**2**

Gyn age youngest tertile

n = 15

Age

youngest tertile

n = 13
