# Supplementary material for: The adolescent transition under energetic stress: Body composition tradeoffs among adolescent women in The Gambia
Source: Evol Med Public Health. 2013 Apr 9;2013(1):75–85. doi: 10.1093/emph/eot005 (PMC3868354; doi:10.1093/emph/eot005)
Supplement: Supplementary Data [file supp_eot005_Reiches_et_al_EMPH_Suppl_Fig_3.docx]

Supplementary Figure 3. Two theoretical pubertal linear growth spurts aligned on peak height velocity. While menarche tends to occur within a year of peak height velocity, the magnitude and duration of spurts in height velocity vary across adolescents. Therefore, time since menarche means different things for different individuals’ relative investments in growth and reproductive function.

menarche

peak height

velocity

time

height velocity
